# Isolated adrenocorticotropic hormone deficiency development during chemotherapy for gastric cancer: a case report

**DOI:** 10.1186/1752-1947-8-90

**Published:** 2014-03-05

**Authors:** Jun Kinoshita, Shinnosuke Higashino, Sachio Fushida, Katsunobu Oyama, Toshifumi Watanabe, Koichi Okamoto, Keishi Nakamura, Hiroyuki Takamura, Itasu Ninomiya, Hirohisa Kitagawa, Takashi Fujimura, Tetsuo Ohta

**Affiliations:** 1 Department of Gastroenterologic Surgery, Division of Cancer Medicine, Graduate School of Medical Science, Kanazawa University, 13-1 Takaramachi, Kanazawa 920-8641, Japan

**Keywords:** Chemotherapy, Gastric cancer, Hyponatremia, Isolated ACTH deficiency

## Abstract

**Introduction:**

Isolated adrenocorticotropic hormone deficiency is an endocrinological disorder characterized by loss of adrenocorticotropic hormone and resultant adrenal insufficiency. Affected patients often present with fatigue, anorexia, and hyponatremia. Although the number of reported cases has been recently increasing, isolated adrenocorticotropic hormone deficiency combined with malignant neoplasia is very rare. Here we describe a patient with gastric cancer who developed unexpected isolated adrenocorticotropic hormone deficiency during chemotherapy.

**Case presentation:**

A 72-year-old Japanese man was admitted to our hospital because of febrile neutropenia due to chemotherapy for gastric cancer recurrence. Although the neutropenia and fever immediately improved, he became unable to take any oral medications and was bedridden 1 week after admission. His serum sodium level abruptly decreased to 122mEq/L on the fifth day of hospitalization. We performed endocrinological studies to investigate the cause of his hyponatremia and plasma hyposmolality. His plasma adrenocorticotropic hormone and cortisol levels were very low. However, his serum levels of all other anterior pituitary hormones were slightly elevated. We then performed a corticotropin-releasing hormone test, which showed that neither his plasma adrenocorticotropic hormone nor cortisol level responded to corticotropin-releasing hormone stimulation. We definitively diagnosed isolated adrenocorticotropic hormone deficiency based on these findings. Hydrocortisone replacement therapy was begun at 20mg/day, resulting in a marked improvement in his anorexia and general fatigue within a few days. His serum sodium level was also normalized immediately after the administration of hydrocortisone. He was discharged from our hospital on the 50th day of hospitalization.

**Conclusions:**

The present case is the second report of a patient with concurrent isolated adrenocorticotropic hormone deficiency and gastric cancer and the first report of a patient diagnosed with isolated adrenocorticotropic hormone deficiency during the course of chemotherapy for a solid malignant neoplasm. Although the symptoms and signs described in the present report are common observations during chemotherapy, it is important to consider not only the adverse effects of antineoplastic agents, but also isolated adrenocorticotropic hormone deficiency as a differential diagnosis. Hydrocortisone replacement therapy for isolated adrenocorticotropic hormone deficiency effectively avoids the unnecessary cessation of chemotherapy.

## Introduction

Adrenocorticotropic hormone (ACTH) is released from the anterior pituitary gland and is an important hormone of the hypothalamic–pituitary–adrenocortical axis. Isolated ACTH deficiency (IAD) was first reported by Steinberg *et al.* in 1954 [1]. The decline in production and secretion of ACTH causes chronic secondary adrenocortical insufficiency, and affected patients often present with anorexia, general fatigue, unconsciousness, and hyponatremia. The diagnosis of IAD usually follows manifestation of adrenal insufficiency under stress, but is hindered by the complete lack of specific symptoms and paucity of measurable signs.

Although the number of diagnosed cases of IAD is increasing because knowledge of the disease and performance of detailed endocrinology testing have become more widespread [2], IAD in combination with malignant neoplasia is very rare [3,4]. Here we describe a patient with gastric cancer who developed unexpected IAD during chemotherapy.

## Case presentation

A 72-year-old Japanese man underwent distal gastrectomy with D2 lymph node dissection for advanced gastric cancer 3 years ago. According to the Union for International Cancer Control tumor, node, metastasis classification, the stage of cancer was T3N2M0 (stage IIIA). After surgery, he received adjuvant chemotherapy with S-1 (TS-1®; Taiho Pharmaceutical Company, Tokyo, Japan) at 120mg/day (4 weeks on, 2 weeks off). Six months after the completion of adjuvant chemotherapy, follow-up computed tomography (CT) revealed ascites and enlarged para-aortic lymph nodes, and his serum carcinoembryonic antigen level was elevated at 10.9ng/mL. Staging laparoscopy was then performed and revealed peritoneal metastasis of his gastric cancer. Then, weekly paclitaxel (Taxol®; Bristol-Myers Squibb, New York, NY, USA) at 80mg/m^2^ (3 weeks of administration followed by 1 week of withdrawal) was begun as first-line treatment of the peritoneal and lymph node recurrence. After four courses of chemotherapy, his serum carcinoembryonic antigen level normalized and CT showed reduction of his para-aortic lymph nodes and disappearance of the ascites.

He developed general fatigue and anorexia soon after completion of seven courses of the above-described chemotherapy. He was then transferred to the out-patient department for a thorough examination. On admission, he was alert with a blood pressure of 110/53mmHg, heart rate of 64 beats per minute, and high body temperature of 38.5°C. His height was 178.5cm, body weight was 60.2kg, and body mass index was 22.3kg/m^2^. No pigmentation was noted in his skin or oral mucosa. The pubic and axillary hair were normal. No abnormal findings were elicited by the chest and abdominal physical examination.

Hematological examination showed leukopenia (white blood cell count, 1.97 × 10^3^/μL), neutropenia (neutrophil count, 350/μL), a hemoglobin level of 11.4g/dL, and a platelet level of 199 × 10^3^/μL. Biochemical examination showed a slightly elevated C-reactive protein level (3.1mg/dL) and a normal blood glucose level (101mg/dL). His serum electrolyte levels were normal at the time of admission (Table 1). Chest and abdominal CT showed no progression of the lymph node metastasis or ascites and no obvious focus of infection.

**Table 1:** Laboratory data on admission

WBC	1970	/μL	ALP	107	IU/L
Neutrophil	350	/μL	γ-GTP	9	IU/L
RBC	404 × 10^4^	/μL	AST	26	IU/L
Hb	11.4	g/dL	ALT	11	IU/L
Bt	35.5	%	LDH	187	IU/L
P1t	19.9 × 10^4^	/μL	Amy	31	IU/L
			T-bil	0.8	mg/dL
BUN	5	mg/dL	TP	6.0	g/dL
Cre	0.66	mg/dL	Alp	3.6	g/dL
Na	141	mEq/L	CRP	3.1	mg/dL
K	4.1	mEq/L	Glucose	101	mg/dL
Cl	101	mEq/L			
			CEA	2.7	ng/mL
			CA19-9	14	U/mL
			CA125	27	U/mL

Abbreviations: *γ-GTP* γ-glutamyltransferase, *ALP* alkaline phosphatase, *ALT* alanine aminotransferase, *Amy* amylase, *AST* aspartate aminotransferase, *BUN* blood urea nitrogen, *CA125* cancer antigen 125, *CA19-9* carbohydrate antigen 19-9, *CEA* carcinoembryonic antigen, *Cl* chlorine, *Cre* creatinine, *CRP* C-reactive protein, *Hb* hemoglobin, *K* potassium, *LDH* lactate dehydrogenase, *Na* sodium, *Plt* platelet, *RBC* red blood cell, *T-bil* total-bilirubin, *TP* total protein, *WBC* white blood cell.

He was admitted to our hospital with a diagnosis of febrile neutropenia. The neutropenia was immediately improved by the administration of granulocyte colony-stimulating factor, and his fever decreased 3 days after admission. By contrast, his anorexia and general fatigue gradually worsened. He was unable to take any oral medications and became bedridden. One week after admission, his Eastern Cooperative Oncology Group performance status deteriorated from 2 to 4. In addition, on the fifth day of hospitalization, his serum sodium level abruptly decreased to 122mEq/L, serum osmolality was 239mOsm/kg, and serum creatinine was 0.69mg/dL. His urine osmolality (482mOsm/kg) was greater than that of plasma (Table 2). We initially restricted his fluid intake to 1000mL/day because we suspected syndrome of inappropriate secretion of antidiuretic hormone (SIADH) as the cause of the hyponatremia. However, his serum sodium level did not normalize despite these treatments. We concurrently performed endocrinological studies in the rest position to investigate the cause of the hyponatremia and plasma hyposmolality. As shown in Table 3, his early morning plasma ACTH level (<5pg/mL) and cortisol level (4.6μg/mL) were very low. However, the plasma levels of all other anterior pituitary hormones, including follicle-stimulating hormone, luteinizing hormone, prolactin, thyroid-stimulating hormone, and growth hormone, were slightly elevated. His serum-free triiodothyronine and serum-free thyroxine levels were also normal. Because the endocrinological findings led us to suspect that his hyponatremia and plasma hyposmolality were caused by ACTH deficiency, we performed a acorticotropin-releasing hormone (CRH) test, which showed that his plasma ACTH and cortisol levels did not respond to CRH stimulation. Antipituitary, antithyroid peroxidase antibodies, and antinuclear antibodies were negative in the immunological examinations.

**Table 2:** Laboratory data during hospitalization

**Blood**			**Urine**		
Na	122	mEq/L	Urine osmolality	482	mOsm/L
Serum osmolality	239	mOsm/L	Na	198	mEq/L
Cre	0.70	mg/dL	Cre	80	mg/dL
			FE_Na_	0.014	%

Abbreviations: *Cre* creatinine, *FE*_*Na*_ fractional excretion of sodium, *Na* sodium.

**Table 3:** Hormonal laboratory data

			**Reference range**				**Reference range**
ACTH	<5.0	pg/mL	(7.4–55.7)	FT3	1.6	pg/mL	(2.4–4.3)
TSH	4.63	μU/mL	(0.24–3.70)	FT4	1.0	ng/dL	(0.9–1.8)
LH	23.2	mIU/mL	(1.22–7.05)	TPO ab	<10.0	IU/mL	(<28.0)
FSH	31.1	mIU/mL	(2.00–8.30)	Tg ab	5.1	IU/mL	(<16.0)
GH	3.23	ng/mL	(<0.42)				
PRL	44.38	ng/mL	(3.58–12.78)	Urinary cortisol	Not detected	μg/day	(11–80)
Renin	2.1	pg/mL	(0.3–2.9)	Antipituitary antibody		(−)	(−)
Somatomedin C	68	ng/mL	(42–250)				
Cortisol	0.4	μg/dL	(4.0–18.3)				
DHEA-S	10	ng/mL	(140–2240)				
Aldosterone	59.0	pg/mL	(29.9–159)				
CRH* test (human corticorelin 100μg i.v.)							
	Minutes	0	15	30	60	90	120
	ACTH (pg/mL)	5.3	5.8	5.6	6.0	5.1	5.4
	Cortisol (μg/dL)	<1.0	<1.0	<1.0	<1.0	<1.0	<1.0

Abbreviations: *ACTH* adrenocorticotropic hormone, *CRH* corticotropin-releasing hormone, *DHEA-S* dehydroepiandrosterone sulfate, *FSH* follicle-stimulating hormone, *FT3* free triiodothyronine, *FT4* free thyroxine, *GH* growth hormone, *i.v.* intravenous, *LH* luteinizing hormone, *PRL* prolactin, *Tg ab* thyroglobulin antibody, *TPO ab* thyroid peroxidase antibody, *TSH* thyroid-stimulating hormone.

**Figure 1:**
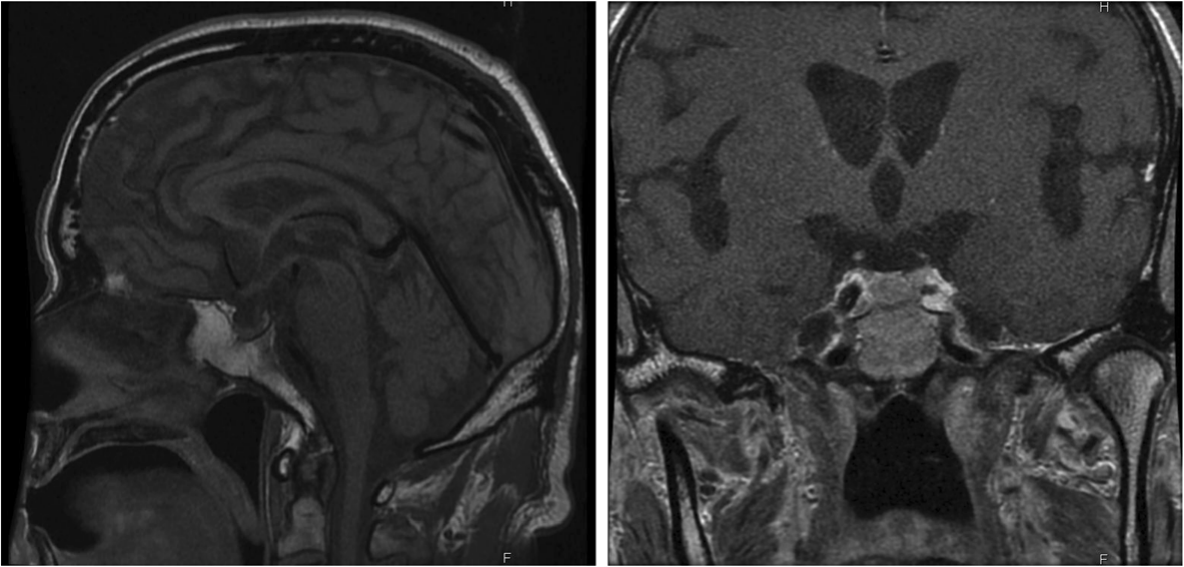
**Magnetic resonance imaging after the onset of isolated adrenocorticotropic hormone deficiency.** Magnetic resonance imaging showed no space-occupying lesions in the pituitary gland or hypothalamus.

CT and magnetic resonance imaging showed no space-occupying lesion or atrophic change in his pituitary gland or hypothalamus (Figure 1).

**Figure 2:**
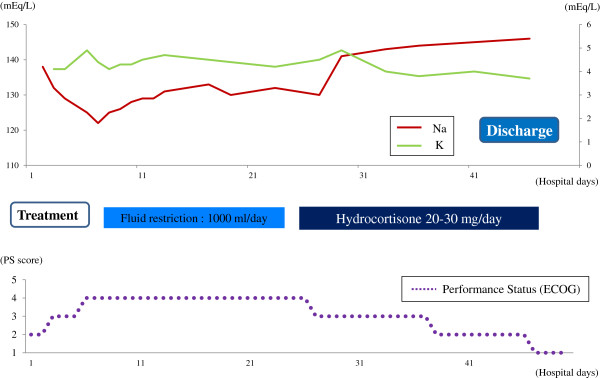
**Clinical course and response to therapy.** The administration of hydrocortisone dramatically improved the patient’s hyponatremia and performance status. Abbreviations: ECOG, Eastern Cooperative Oncology Group; K, potassium; Na, sodium; PS, performance status.

Because we definitively diagnosed IAD based on these findings, hydrocortisone replacement therapy was commenced at 20mg/day and gradually increased to 30mg/day, resulting in a marked improvement of his anorexia and general fatigue within a few days. His serum sodium level was also normalized immediately after the administration of hydrocortisone. His performance status returned to the prehospital state, and he was discharged from our hospital on the 50th day of hospitalization (Figure 2). He then underwent out-patient chemotherapy with hydrocortisone replacement. He remains alive without progression of the metastatic lesions 6 months after discharge.

## Discussion

We have described a case of IAD in a patient with gastric cancer undergoing chemotherapy. He was diagnosed based on a decrease in his ACTH and cortisol blood concentrations and the lack of adequate response of ACTH and cortisol levels to CRH administration.

Although the number of diagnosed cases of IAD is increasing, patients with concurrent IAD and malignant neoplasms are very rare. Only one patient with acute lymphoblastic leukemia and one with gastric cancer have been previously reported [3,4]. Kamiya and Murakami reported a type 2 diabetic patient with concurrent IAD and gastric cancer who was positive for antipituitary antibody and had empty sella [4]. To the best of our knowledge, the present case is the second report of a patient with concurrent IAD and gastric cancer and the first report of a patient diagnosed as IAD during the course of chemotherapy for a solid malignant neoplasm.

Although the etiology of IAD remains uncertain in most cases, the main causes of IAD are considered to be autoimmune processes, congenital and genetic effects, incomplete infarction of the pituitary gland after delivery, and hypothalamic lesions secondary to birth trauma or head injury. IAD has been especially well described in combination with various autoimmune disorders, such as Graves’ disease [5], Crohn’s disease [6], Hashimoto’s thyroiditis [7], myasthenia gravis [8], and type 1 diabetes mellitus [9]. In addition, 48% of patients with IAD were positive for antipituitary antibody in one report [10]. However, the present patient was neither positive for antithyroid or antipituitary antibody nor had a medical or family history of these autoimmune disorders. In addition, findings of brain CT and pituitary magnetic resonance imaging showed no abnormality such as a tumor, infarction, trauma, or inflammation.

However, it has been reported that powerful stressors such as external wounds, infections, surgery, or bleeding can trigger acute adrenal insufficiency [11]. Although it is difficult to clarify whether the gastric cancer metastasis or chemotherapy triggered the onset of IAD in our case, we speculate that the stress of febrile neutropenia, which was a severe adverse effect of chemotherapy, might have triggered the onset of IAD because the patient had undergone gastric cancer treatment without IAD symptoms for more than 2 years.

Nausea, vomiting, and general fatigue are the most common symptoms of adrenal insufficiency and are seen in more than 90% of patients [12]. Hyponatremia without hyperkalemia is regarded as one of the clinical features of IAD [13]. These symptoms of IAD are quite similar to the adverse effects of antineoplastic agents; therefore, the diagnosis of IAD was difficult in the present case. Thus, it is possible that a substantial number of patients with undiagnosed IAD undergo chemotherapy because the clinical features of IAD are similar to the adverse effects of chemotherapy.

Hyponatremia is rare in patients with central adrenocortical insufficiency because the sodium balance is mainly regulated by the renin-angiotensin system. However, it is well known that longstanding chronic adrenal insufficiency without hydrocortisone replacement induces impaired mineralocorticoid secretion. In general, SIADH is the most probable cause of hyponatremia and hyposmolality due to antineoplastic agents. It is important to distinguish SIADH from other endocrine disorders, especially adrenal insufficiency, in patients undergoing chemotherapy.

Hyponatremia in patients with adrenal insufficiency may occur as a result of the inability to excrete a water load, a syndrome that resembles SIADH [14]. Indeed, we initially suspected SIADH and restricted the patient’s fluid intake, which was not effective in the treatment of hyponatremia. Instead, the administration of hydrocortisone after the diagnosis of IAD immediately improved not only his hyponatremia, but also his generalized fatigue and anorexia. Although these symptoms and signs are common observations during chemotherapy, it is important to consider not only the adverse effects of antineoplastic agents, but also IAD as a differential diagnosis. Hydrocortisone replacement for the treatment of IAD effectively avoids the unnecessary cessation of chemotherapy.

## Conclusions

In conclusion, we have described a patient with gastric cancer who was diagnosed with IAD during the course of chemotherapy. To the best of our knowledge, this is the first report of IAD in relation to chemotherapy for a solid cancer.

## Consent

Written informed consent was obtained from the patient for publication of this case report and accompanying images. A copy of the written consent is available for review by the Editor-in-Chief of this journal.

## Abbreviations

ACTH: Adrenocorticotropic hormone; CT: Computed tomography; CRH: Corticotropin-releasing hormone; IAD: Isolated ACTH deficiency; SIADH: Syndrome of inappropriate secretion of antidiuretic hormon.

## Competing interests

The authors declare that they have no competing interests.

## Authors’ contributions

JK wrote the case report. SF and TF interpreted the data relating to the oncologic disease. JK, SH, TW, KoO, KN and KaO performed the physical examination and medical care. HT, IN, HK, and TO contributed to the writing and revision of the manuscript. All authors read and approved the final manuscript.

## References

[B1] Steinberg A Shechter FR Segal HI True pituitary Addison’s disease, a pituitary unitropic deficiency; fifteen-year follow-up J Clin Endocrinol Metab 1954 14 1519 1529 10.1210/jcem-14-12-1519 13211787

[B2] Yamamoto T Kamoi K Prevalence of maturity-onset isolated ACTH deficiency (IAD) in 2005: Japanese cohort studies Endocr J 2008 55 939 941 10.1507/endocrj.K08E-146 18552459

[B3] Yamaguchi H Nakamura H Mamiya Y Yamamoto Y Tajika K Sugihara H Gomi S Inokuchi K Hasegawa S Shibazaki T Dan K Wakabayashi I Acute lymphoblastic leukemia with isolated adrenocorticotropic hormone deficiency Intern Med 1997 36 819 821 10.2169/internalmedicine.36.819 9392357

[B4] Kamiya Y Murakami M Type 2 diabetes mellitus accompanied by isolated adrenocorticotropic hormone deficiency and gastric cancer Intern Med 2009 48 1031 1035 10.2169/internalmedicine.48.1972 19525593

[B5] Miyauchi S Yamashita Y Matsuura B Onji M Isolated ACTH deficiency with Graves’ disease: a case report Endocr J 2004 51 115 119 10.1507/endocrj.51.115 15004417

[B6] Kalambokis G Vassiliou V Vergos T Christou L Tsatsoulis A Tsianos EV Isolated ACTH deficiency associated with Crohn’s disease J Endorinol Invest 2004 27 961 964 10.1007/BF03347541 15762046

[B7] Gürlek A Nar A Gedik O Isolated adrenocorticotropic hormone deficiency, thyroid autoimmunity, and transient hyperprolactinemia Endocr Pract 2001 7 102 105 10.4158/EP.7.2.102 11421554

[B8] Corcuff JB Lafranque P Henry P Roger P Isolated corticotroph insufficiency associated to myasthenia gravis J Endocrinol Invest 1997 20 669 671 9492106 10.1007/BF03348029

[B9] Sakane N Yoshida T Yoshioka K Umekawa T Kondo M Severe hypoglycemia and type I diabetes with isolated ACTH deficiency Diabetes Care 1995 18 1621 1622 8722062 10.2337/diacare.18.12.1621

[B10] Sugiura M Hashimoto A Shizawa M Tsukada M Maruyama S Ishido T Kasahara T Hirata T Heterogeneity of anterior pituitary cell antibodies detected in insulin-dependent diabetes mellitus and adrenocorticotropic hormone deficiency Diabetes Res 1986 3 111 114 3011344

[B11] de Luis DA Aller R Romero E Isolated ACTH deficiency Horm Res 2008 49 247 249 10.1159/000023179 9568810

[B12] Burke CW Adrenocortical insufficiency Clin Endocrinol Metab 1985 14 947 976 10.1016/S0300-595X(85)80084-0 3002680

[B13] Hannon MJ O’Halloran DJ Isolated acquired ACTH deficiency and primary hypothyroidism: a short series and review Pituitary 2011 14 358 361 10.1007/s11102-008-0164-9 19110973

[B14] Levinsky NG Isselbacher KJ, Braunward E, Wilson JD, Martin JB, Fauci AS, Kasper DL Fluids and electrolytes Harrison’s Principles of Internal Medicine 1994 13 St Louis: McGraw-Hill 246

